# White blood cell detection, classification and analysis using phase imaging with computational specificity (PICS)

**DOI:** 10.1038/s41598-022-21250-z

**Published:** 2022-11-21

**Authors:** Michae J. Fanous, Shenghua He, Sourya Sengupta, Krishnarao Tangella, Nahil Sobh, Mark A. Anastasio, Gabriel Popescu

**Affiliations:** 1 Quantitative Light Imaging Laboratory, Beckman Institute for Advanced Science and Technology, University of Illinois at Urbana-Champaign, Urbana, IL 61801 USA; 2 Department of Bioengineering, University of Illinois at Urbana-Champaign, 306 N. Wright Street, Urbana, IL 61801 USA; 3 Department of Computer Science and Engineering, Washington University in St. Louis, 1 Brookings Drive, St. Louis, MO 63130 USA; 4 Department of Electrical and Computer Engineering, University of Illinois at Urbana-Champaign, 306 N. Wright Street, Urbana, IL 61801 USA; 5 Christie Clinic, 1400 West Park Street, Urbana, IL 61801 USA; 6 NCSA Center for Artificial Intelligence Innovation, University of Illinois at Urbana-Champaign, Urbana, IL 61801 USA; 7 Department of Computer Science, University of Illinois at Urbana-Champaign, Urbana, IL 61801 USA

**Keywords:** Biological techniques, Imaging, Optical imaging

## Abstract

Treatment of blood smears with Wright’s stain is one of the most helpful tools in detecting white blood cell abnormalities. However, to diagnose leukocyte disorders, a clinical pathologist must perform a tedious, manual process of locating and identifying individual cells. Furthermore, the staining procedure requires considerable preparation time and clinical infrastructure, which is incompatible with point-of-care diagnosis. Thus, rapid and automated evaluations of unlabeled blood smears are highly desirable. In this study, we used color spatial light interference microcopy (cSLIM), a highly sensitive quantitative phase imaging (QPI) technique, coupled with deep learning tools, to localize, classify and segment white blood cells (WBCs) in blood smears. The concept of combining QPI label-free data with AI for the purpose of extracting cellular specificity has recently been introduced in the context of fluorescence imaging as phase imaging with computational specificity (PICS). We employed AI models to first translate SLIM images into brightfield micrographs, then ran parallel tasks of locating and labelling cells using EfficientNet, which is an object detection model. Next, WBC binary masks were created using U-net, a convolutional neural network that performs precise segmentation. After training on digitally stained brightfield images of blood smears with WBCs, we achieved a mean average precision of 75% for localizing and classifying neutrophils, eosinophils, lymphocytes, and monocytes, and an average pixel-wise majority-voting F1 score of 80% for determining the cell class from semantic segmentation maps. Therefore, PICS renders and analyzes synthetically stained blood smears rapidly, at a reduced cost of sample preparation, providing quantitative clinical information.

## Introduction

White blood cells (WBCs) are essential components of the immune system and of the body's protection against infections. The five key forms of WBCs are lymphocytes (including B and T cells), eosinophils, neutrophils, monocytes and basophils. In healthy individuals, these WBC populations have specific concentration ranges and any deviations from these parameters are clinically informative^1^.

Wright's staining of blood smears is one of the most common methods for detecting white blood cell aberrations^2^. However, it is time-consuming and laborious for a clinical pathologist to detect and identify individual cells in order to diagnose leukocyte abnormalities. Furthermore, the staining procedure is involved, which altogether makes diagnosis tied to a clinical infrastructure.

Another way to calculate WBC relative percentages is with flow cytometry, where fluorescently labeled antibodies are used to differentially mark WBC populations^3^. With long-established laboratory procedures, this approach is widely used in clinical practice. Modern cytometers, such as mass cytometers, can evaluate up to 40 parameters in any single measurement. However, they are limited to analyzing cell phenotypes based on the expression degree of antibody labels, similar to fluorescence-based cytometers^4^.

Recent efforts to further facilitate these tasks by analyzing label-free white blood cells include the use of intensity-based imaging flow cytometry^3,5^, in which the benefits of digital microscopy, such as the measurement of morphology, are combined with the high-throughput and statistical certainty of a flow cytometer. Using this technique, white blood cells can be detected, classified and counted. However, this method requires expensive equipment, involved sample preparation, is of low resolution, and does not provide quantitative information on cellular components.

Quantitative phase imaging (QPI)^6–25^ is a label-free imaging method that can evaluate pathlength changes in biological samples at the nanometer scale. QPI has a variety of medical diagnostic applications^26^ : Di Caprio et al. have applied QPI to study sperm morphology^27^ ; Marquet et al. have used QPI to examine living neurons^28^; Lee et al. used it to investigate cell pathophysiology^29^; and Din et al. have used it to perform research on macrophages and hepatocytes^30^.

Phase imaging approaches typically use coherent light sources, which compromises multiple factors of image quality, such as signal-to-noise ratio (SNR) and contrast, due to speckles. SLIM overcomes this drawback by using a broadband field to derive nanoscale details and dynamics in live cells using interferometry^31^. We previously used color spatial light interference microscopy (cSLIM) to examine piglet brain tissue^32,33^. This system uses a brightfield objective and an RGB camera, and generates 4 intensity images, a regular color micrograph being one of them. Thus, cSLIM simultaneously produces both a brightfield image and a phase map. This image, φ(x,y), is a data matrix relating to the nanoarchitecture of the imaged sample.

There has recently been a surge of interest in using AI to analyze relevant datasets in medical fields^34–41^. AI has unique image processing capabilities allowing it to detect multi-dimensional features that qualified pathologists would otherwise miss. Deep convolutional networks enable thousands of image-related feature sets to be tested to recognize complex biological data^42,43^.

Here, we apply phase imaging with computational specificity (PICS)^44–46^, a novel microscopy technique that combines AI computation with quantitative data, to analyze WBCs in blood smears. Specifically, we combine deep learning networks with cSLIM micrographs to detect, classify and segment four types of white bloods cells: neutrophils, lymphocytes, monocytes, and eosinophils. To the best of our knowledge, this is the first time such a strategy has been implemented. Such a system does not require staining of cells as we convert phase maps, which contain biologically relevant data into Wright’s stain brightfield images. This is unprecedented and indisputably valuable for standard clinical produces requiring the accurate assessment of WBCs without the use of tedious preparations and extraneous labels.

## Methods

### Phase imaging with computational specificity

**Figure 1:**
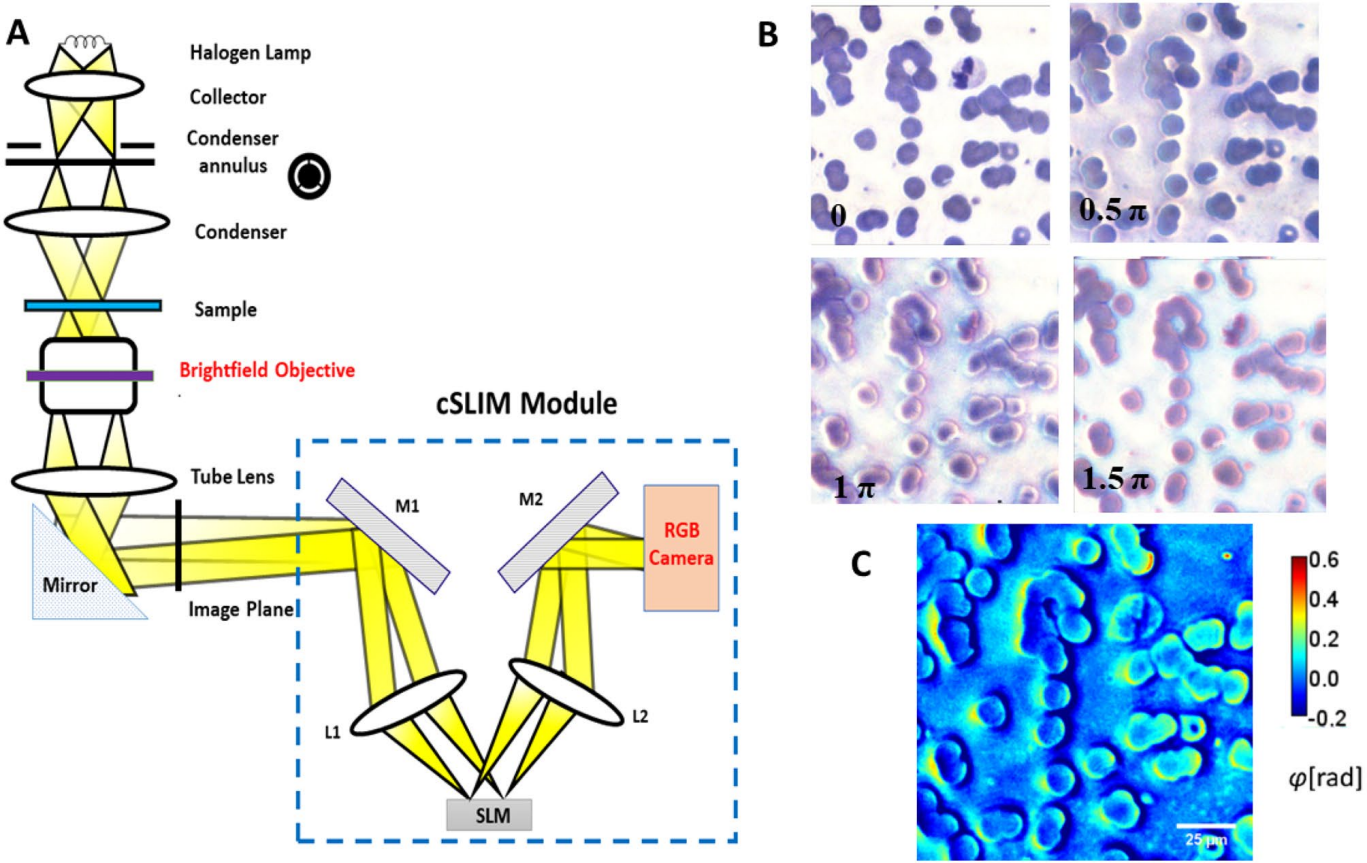
Schematic setup for cSLIM. (**A**) The cSLIM module is attached to a commercial phase contrast microscope and uses a brightfield objective with an RGB camera. (**B**) The four phase-shifted color interferograms, with the initial unshifted frame corresponding to a brightfield image. (**C**) Computed SLIM phase image.

Our label-free SLIM scanner comprises custom hardware and in-house developed software. The cSLIM principle of operation relies on phase shifting interferometry applied to a phase contrast setup (see Ref.^31^ for details). We shift the phase delay between the incident and scattered field in increments of π/2 and acquire 4 respective intensity images, which is sufficient to compute the phase image unambiguously. Figure 1A shows the optical diagram of the cSLIM system. Figure 1B and C show an example of the four phase-shifted color frames and extracted quantitative image of a blood smear, respectively. Using our in-house software and the traditional ‘stop-and-stare’ scanning method, we acquire an individual frame in roughly 0.25 s, and capture a scan of 625 frames in 10 min. The samples used here are fixed blood smears and therefore don’t move regardless of acquisition speed.

It should be noted that the cSLIM setup does not remove the halo artifact, as each of the four frames is acquired with the ring illumination. This artifact is therefore still present in the final quantitative map, causing fake shading around low frequency features.

### Blood smears preparation

**Figure 2:**
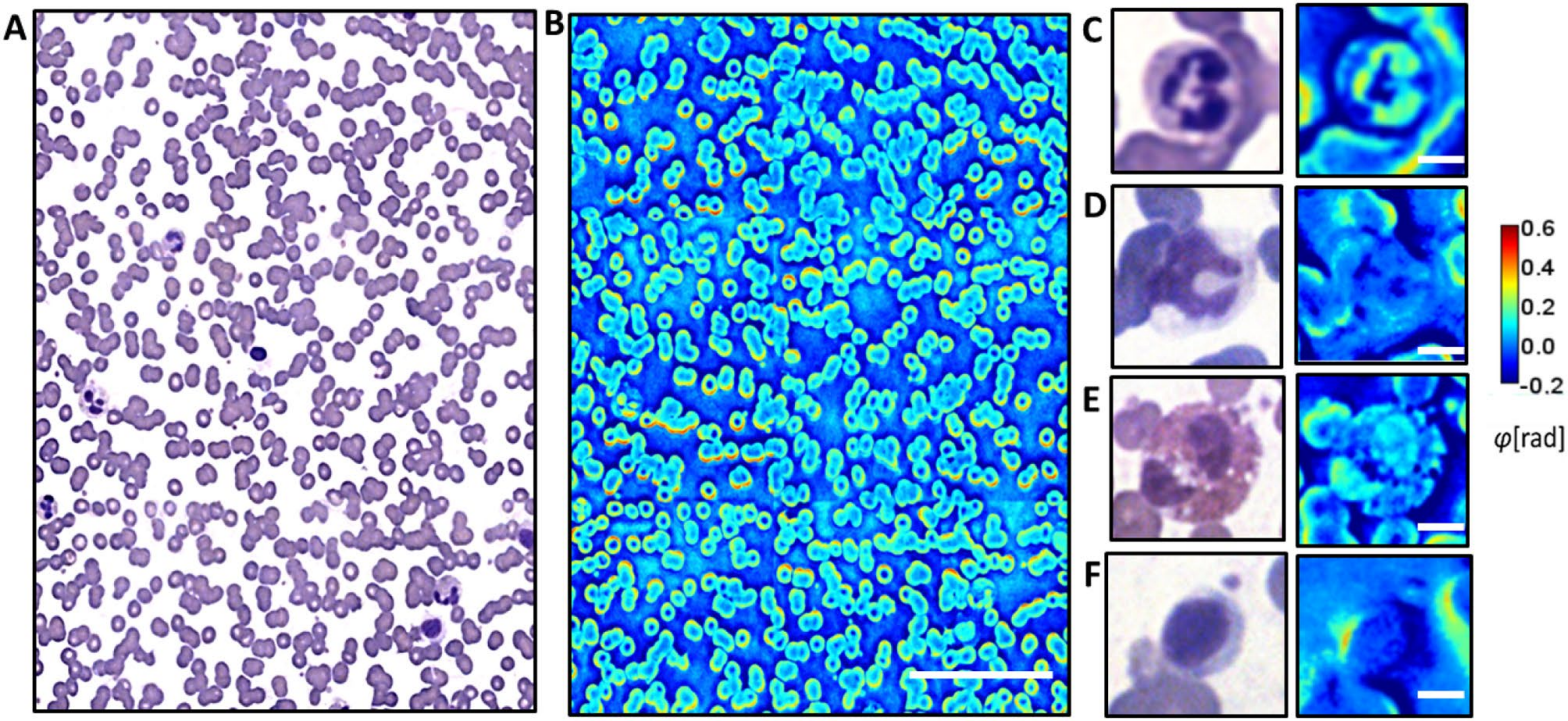
Blood smear images. (**A**) 3 × 4 stitch of brightfield images from a single scan. Scale bar: 200 µm. (**B**) corresponding SLIM images to (**A**). Examples of (**C**) a neutrophil, (**D**) monocyte, (**E**) eosinophil, and (**F**) lymphocyte in brightfield and phase channels. Scale bars: 5 µm.

18 blood smears stained with Wright’s stain were used for our analysis. Each smear was scanned in a configuration of 12 × 12–40 frames, depending on the size of the ‘zone of morphology,’ to avoid clumped areas at the application point as well as sparse areas near the feathered edge. Examples of brightfield and counterpart phase images (3 × 4 frame stitches) of the smears are shown in Fig. 2A, B, with zoom-in instances of a neutrophil, monocyte, eosinophil, and lymphocyte in Fig. 2C–F. Each slide was imaged to produce both brightfield and quantitative channels. The classifications of boundaries and segmentations were performed manually in MATLAB using the ‘Image Labeler’ application. Basophils were omitted from analysis due to insufficient numbers needed for training and validation. The ground truth classification of the WBCs was performed by a board-certified hematopathologist. The procedures used in this study for conducting experiments using human subjects were approved by the institute review board at the University of Illinois at Urbana–Champaign (IRB Protocol Number 13900). Furthermore, all blood smear slides came already fully prepared, and all methods were carried out in accordance with relevant guidelines and regulations, and dissociated from patient statistics, with informed consent that was obtained from all subjects and/or their legal guardians.

### Image-to-image translation

The purpose of converting phase images to the original brightfield version is for situations when an unstained slide is imaged or a slide is captured with a grayscale camera. The phase map created with cSLIM is the equivalent of these versions. A translation from QPI to Wright’s stain, as far we know, has never been heretofore achieved.

The conversion of SLIM micrographs to artificial brightfield images of stained WBCs was accomplished using a conditional generative adversarial network (GAN) based network called pix2pix^47^. GAN models have been very successfully at converting micrographs types from one modality to another^34,48^. The pix2pix model was chosen because converting SLIM images to brightfield counterparts is a purely pixel-wise transformation in intensity and retrieving quantitative data is not required. A total of 504 images were used for processing and among these 50 images were held out as test images. The remaining images were split between training and validation in an 8:1 ratio. In this pix2pix network, there are two components: a generator (G) and a discriminator (D). The task of the discriminator is to distinguish between a real image and the fake image generated by the generator. The generator and discriminator play an adversarial min–max game, the GAN loss can be mathematically written as:

1 $$L_{cGAN} (G,D) = E_{x,z} [\log D(x,y)] + E_{x,z} [\log (1 - D(x,G(x,z))]$$

where $E_{x,z}$ is the expected value over real and fake instances, x is the input image, and z is the random noise. This trains the generator *G* to create artificial images which are supposed to fool the discriminator. An L1 loss is also combined with this GAN loss for more stable training. An Adam optimizer^49^ with a learning rate of 0.0002 was used to train the generator and the batch size was set to 2. Input SLIM images were downsampled to 512X512 to fit the GPU memory.

**Figure 3:**
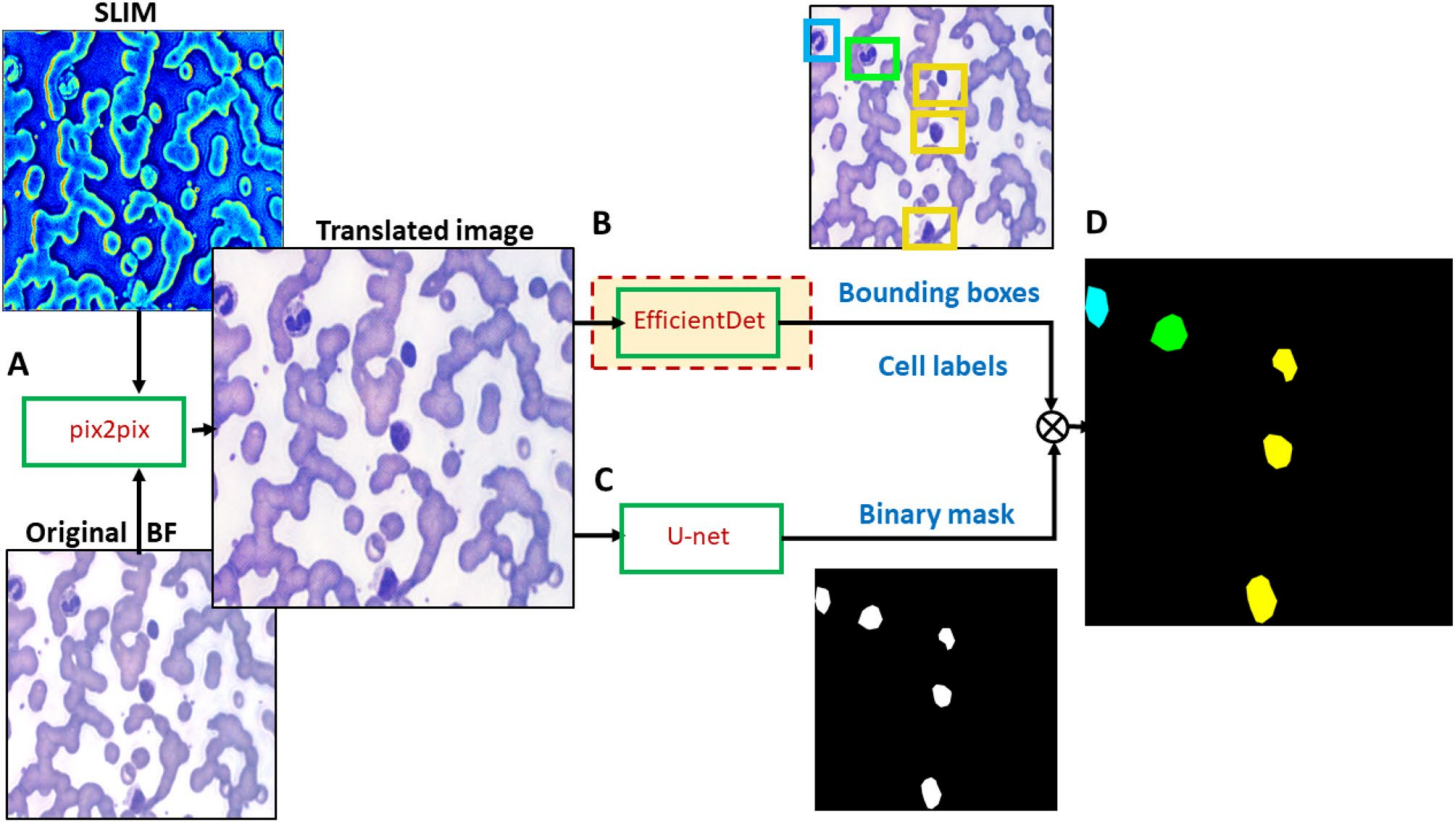
Image processing. (**A**) The procedure for analyzing WBCs begins with an image-to-image translation with pix2pix from SLIM to brightfield. (**B**) The translated image is then trained with EfficientDet to locate and classify all cell types, (**C**) in parallel with a U-net that produces binary masks of the WBCs. (**D**) Finally, combining both networks enables the semantic segmentation of different WBCs in each frame.

The semantic segmentation was performed in multiple steps as shown schematically in Fig. 3. The EfficientDet model^50^ was used for localization and classification of different WBC classes. A U-Net was used to generate binary segmentation maps of WBC cells. The localizations and binary maps were combined to generate semantic segmentation maps through a process described below. The same test images were used in these steps also. The training and validation data were split randomly 5 times, and with each trained model the localization, classification and segmentation was performed with the same set of testing images. The detailed descriptions of these steps are given below.

### Localization and classification of WBCs

The localization and classification of white blood cells were performed by a state-of-the-art deep learning-based object detection model, EfficientDet^50^. The EfficientDet model took an image as input and predicted localization-bounding boxes with associated WBC class labels. An example of a rectangular label is shown in Fig. S1A. In Fig. 3, a generic schematic is shown where the input of the EfficientDet model is a translated brightfield image. In this study, the architecture of the EfficientDet was specified as EfficientDet-D0. It uses EfficientNet-B0 as the backbone network for feature extraction. The weights of EfficientNet-B0 in the EfficientDet model were initialized with an EfficientNet-B0 that was pre-trained on the ImageNet dataset^51^ for an image classification task. The whole EfficientDet network was subsequently fine-tuned by use of the translated images in the training set. In the fine-tuning process, the EfficientDet was trained by minimizing a compound focal and smooth L1 loss that measures the classification errors and bounding box prediction errors, respectively^50^. The loss function was minimized with an Adam optimizer^49^ with a batch size of 8. The learning rate was set to ${5\times 10}^{-5}$, which was determined based on the network performance on the validation set. The network training was stopped if the mean average precision (mAP) of validation set did not increase for 10 consecutive epochs. The EfficientDet network weights that yielded the highest validation mAP during the training process were selected to establish the final EfficientDet model.

The trained EfficientDet was tested on the unseen testing set with mAP as the performance metric. The mean classification and localization time per frame was 140 ms. For a more robust evaluation, the training process described above was repeated five times corresponding to the five random partitions of training and validation data. These five EfficientDet models were tested on the same unseen testing set. The corresponding outcomes are discussed in the *Results* section.

### Binary segmentation of WBCs

For binary segmentation of WBC cells, a U-Net network was used. The U-Net model took a translated brightfield image as input and predicted a binary map in which each pixel represented either WBC class or background class. An example of how cells were labeled for this is shown in Fig. S1B. In Fig. 3, a general schematic is shown where the input to the U-Net model is a translated brightfield image and the output is a binary segmentation map.

The architecture of the U-net consists of 5 blocks and 4 blocks in the expansion path and contraction path, respectively. Each block in the expansion path includes a convolutional layer, Max-pooling layer, and a BatchNorm layer; each block in the contraction path includes a convolutional layer, an up-sampling layer, and a BatchNorm layer.

The training and validation sets used for U-Net were consistent with those used for the previous EfficientDet model training. In this step, the ground truth for each image sample was the binary mask map of WBCs. The U-Net was trained by using an Adam optimizer^49^ to minimize the mean squared error loss that measures the difference between the ground truth segmentation map and the prediction of the U-Net. The learning rate and batch size were ${3\times 10}^{-5}$ and 2, respectively. The validation loss was monitored in the training process. The network training was stopped if there was no decrease in validation loss for 5 consecutive epochs, while we chose the weights corresponding to the lowest validation loss. The training process was repeated five times based on the same partitions of training and validation data described previously. Additionally, the testing dataset was the same as described in the previous section of image-to-image translation, with mean processing times of 110 ms per frame.

### Semantic map generation

In the previous two steps, localization-classification of WBC cells using EfficientDet model and binary segmentation by U-Net model were described. This step combined the results of these previous two steps to generate semantic maps. In Fig. 3, for a given translated brightfield image, the output of the EfficientDet model and U-Net model were combined to generate the semantic segmentation map. The semantic segmentation map was generated by combining the predicted labeled boxes and binary maps in a pixel-wise manner, described as follows: the pixels outside cell regions in the binary map were classified into the background class. For pixels inside cell regions, if they also existed inside one unique labeled box, these pixels were classified into the WBC class of the associated cell box; if a pixel was contained within two or more labeled boxes, which was observed to be a very rare case in our studies, the pixel was classified into the WBC class of the labeled box with the highest confidence score according the majority class; in all other cases, the pixels were assigned to the background class.

With the five pairs of trained EfficientDet and U-Net models in the previous two steps, five semantic maps were generated for a given translated image. The five semantic maps were finally combined into one for a more reliable prediction by applying a pixel-wise majority-voting process. The combined semantic map was the final predicted semantic map for the image. Pixel-wise recall, precision, and F1 scores were used to evaluate the semantic segmentation performance of the proposed approach on the unseen testing set. The pixel-wise recall, precision, and F1 scores were computed between the predicted semantic maps and their ground truth values for all the images in the unseen test. The corresponding results are discussed in the *Results* section.

## Results

Our dataset included 504 images that were selected from the scans to include white blood cells. We analyzed 267 neutrophils, 117 eosinophils, 192 lymphocytes, and 82 monocytes. There were insufficient basophils to include in the set. Due to the natural proportion of white blood cells to one another, with neutrophils comprising 40–60%, lymphocytes 20–40%, monocytes 2–8%, and eosinophils 1–4% of total WBCs ^52,53^, it was difficult to deliberately make an even number of cells in each category. Although the SLIM images contribute new structural information, the color disparity in some of the cellular components is sometimes diminished, such as in WBC nuclei.

**Figure 4:**
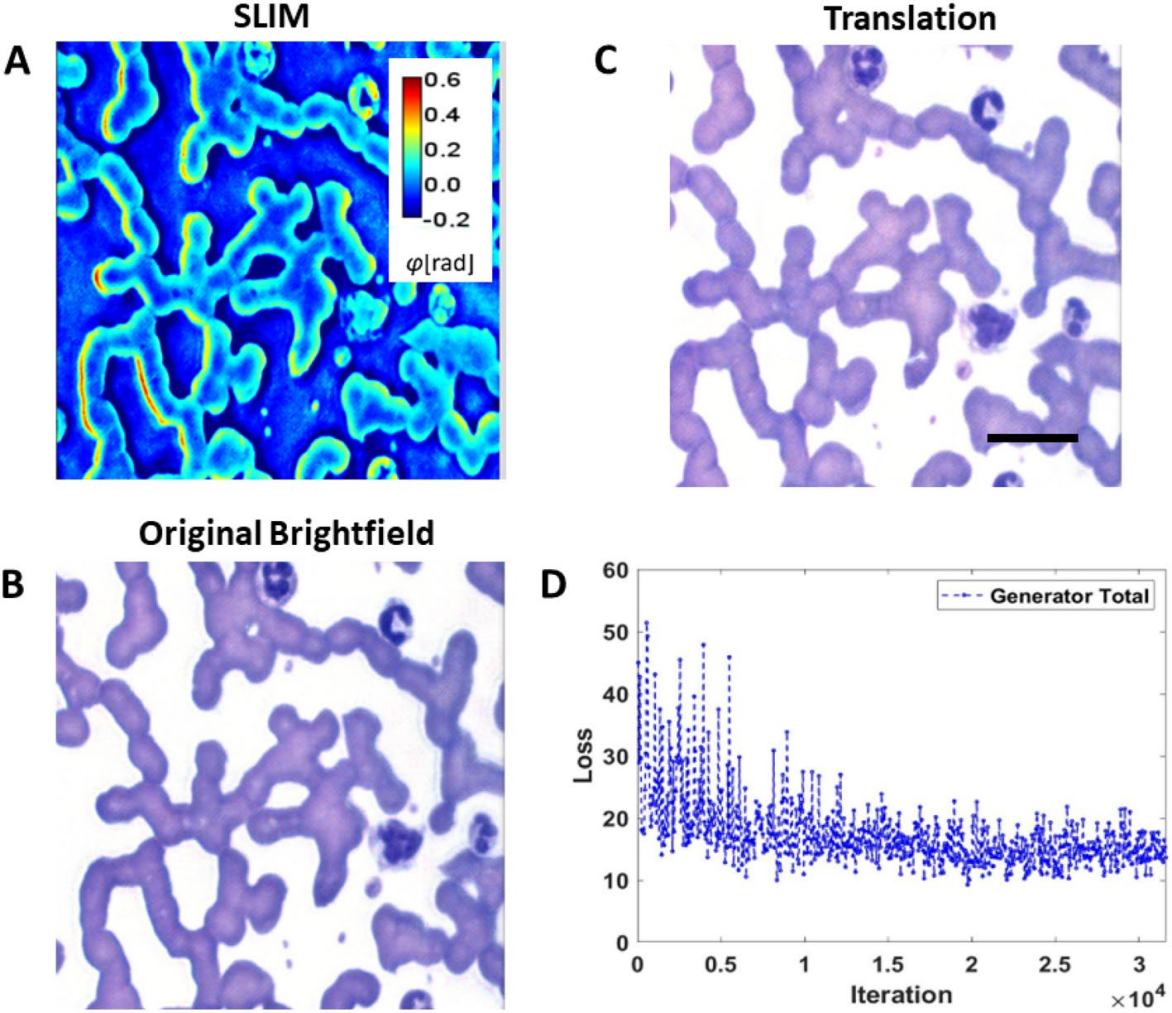
Image-to-image translations. (**A**) Example of an input SLIM micrograph. (**B**) Corresponding brightfield image that serves as ground truth. (**C**) The translated brightfield output of the model. (**D**) Loss plot of the training sessions, combing GAN and L1 losses. Scale bar: 25 µm.

In Fig. 4, a sample translated brightfield image, generated by the image-to-image translation model from an input SLIM image, is shown along with the corresponding original brightfield images. Visually, both the images appear identical. Upon closer inspection, it can be seen that some of the WBC nuclei are not as dark in hue as in the original images, and the background is slightly grainy in some areas. Figure 4D shows the combined GAN and L1 loss plots of the generator. The model weights corresponding to the lowest validation loss were chosen to generate the translated brightfield images from the test set SLIM images. The quantitative results for localization, classification and segmentation are described in the following sections.

### Localization and classification

The mean and standard deviation of mAPs for the four categories (neutrophils, eosinophils, lymphocytes, and monocytes) corresponding to five sets of testing are shown in Table 1. For a comparative analysis, we employed the same strategy to train five EfficientDet models with annotated SLIM images and ground truth brightfield images, respectively. The corresponding results are shown in Table 1.

**Table 1:** Localization and Classification.

Category	Test 1 mAP	Test 2 mAP	Test 3 mAP	Test 4 mAP	Test 5 mAP	Mean mAP
SLIM images	0.7581	0.7543	0.7301	0.7196	0.6696	0.7263 ± 0.032
Brightfield images	0.8476	0.8545	0.8544	0.8736	0.8112	0.848 ± 0.02
Translated images	0.7742	0.7805	0.6877	0.7569	0.7363	0.7469 ± 0.03

Mean average precision (AP) values for localization and classification of all five test sets, using EfficientDet, for SLIM, brightfield and translated images.

**Figure 5:**
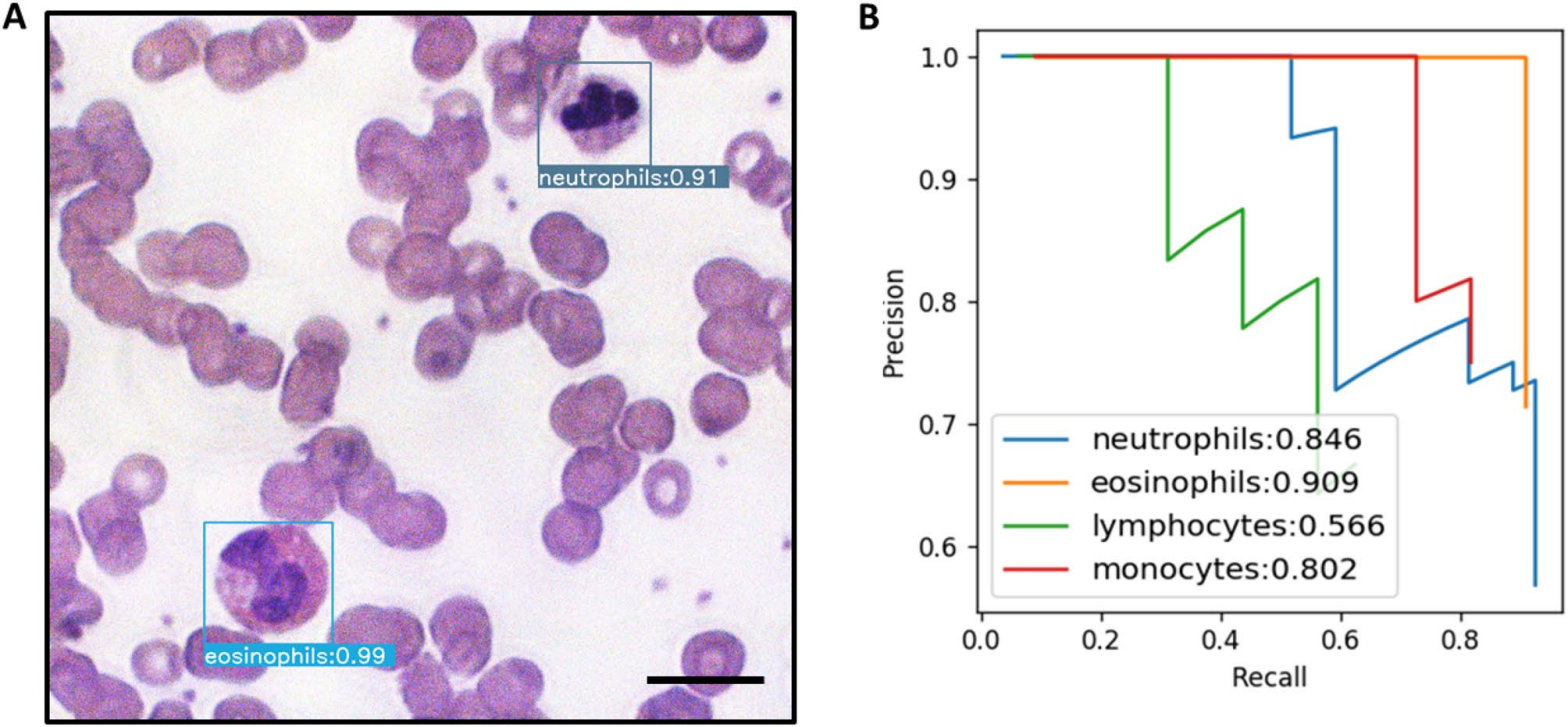
Localization and classification. (**A**) Example of an input translated brightfield micrograph with an eosinophil and neutrophil located and classified with 99% and 91% certainty, respectively. (**B**) Precision-Recall curves for all cell categories in all images. Scale bar: 20 µm.

In general, the brightfield and translated images both produced better results for the four WBC categories in terms of the average mAPs over the five test sets. An example of a labeled translated image is shown in Fig. 5A. In this case, there is an eosinophil in the bottom left corner that is recognized and correctly labeled with 99% certainty. In the top right corner, a neutrophil is correctly labeled with 91% certainty. These values are not indicative of all respective cell types, even in the case of localization and classification, but only pertain to these specific cases. The Precision-Recall curves for each WBC in all tested translated images are shown in Fig. 5B, with comparisons of original image types in Fig. S2. The best performance is that of the eosinophils, with a precision score of 90.09%, likely due to a very distinctive red granular cytoplasm, and the lowest is that of lymphocytes, with 56.6%, likely because many large lymphocytes appear similar to small monocytes, even to a trained pathologist. These translated images were produced using only quantitative phase image input and can therefore be regarded as being equivalent to unlabeled blood smears.

### Segmentation

The results of the semantic segmentation on translated images are listed in Tables 2, 3. These numbers are based on pixel-wise F1 scores. Eosinophils had the highest scores, with 86.7%, and neutrophils and lymphocytes had the lowest, with 68.5%, for reasons similar to those in localization and classification tasks. These results confirm the reliability of the translated model to convert unseen, unstained quantitative phase images into typical Wright’s stain brightfield images.

**Table 2:** Semantic Segmentation.

	Test 1	Test 2	Test 3	Test 4	Test 5	Average
SLIM	0.785	0.769	0.749	0.762	0.709	0.7548 ± 0.026
BF	0.81	0.819	0.82	0.831	0.796	0.8152 ± 0.012
Trans	0.796	0.81	0.749	0.742	0.735	0.7664 ± 0.031

Semantic segmentation results in terms F1 scores for all five test sets, and the average values for all three image versions.

**Table 3:** Majority Voting.

Class	Background	Neutrophils	Eosinophils	Lymphocytes	Monocytes	Average
SLIM	0.996	0.716	0.925	0.591	0.701	0.786
BF	0.997	0.755	0.901	0.696	0.797	0.829
Trans	0.997	0.685	0.867	0.685	0.757	0.798

Results based on the combination of all five semantic maps for a reliable prediction with majority-voting: F1 scores for image and cell classes, with average values in the rightmost column.

**Figure 6:**
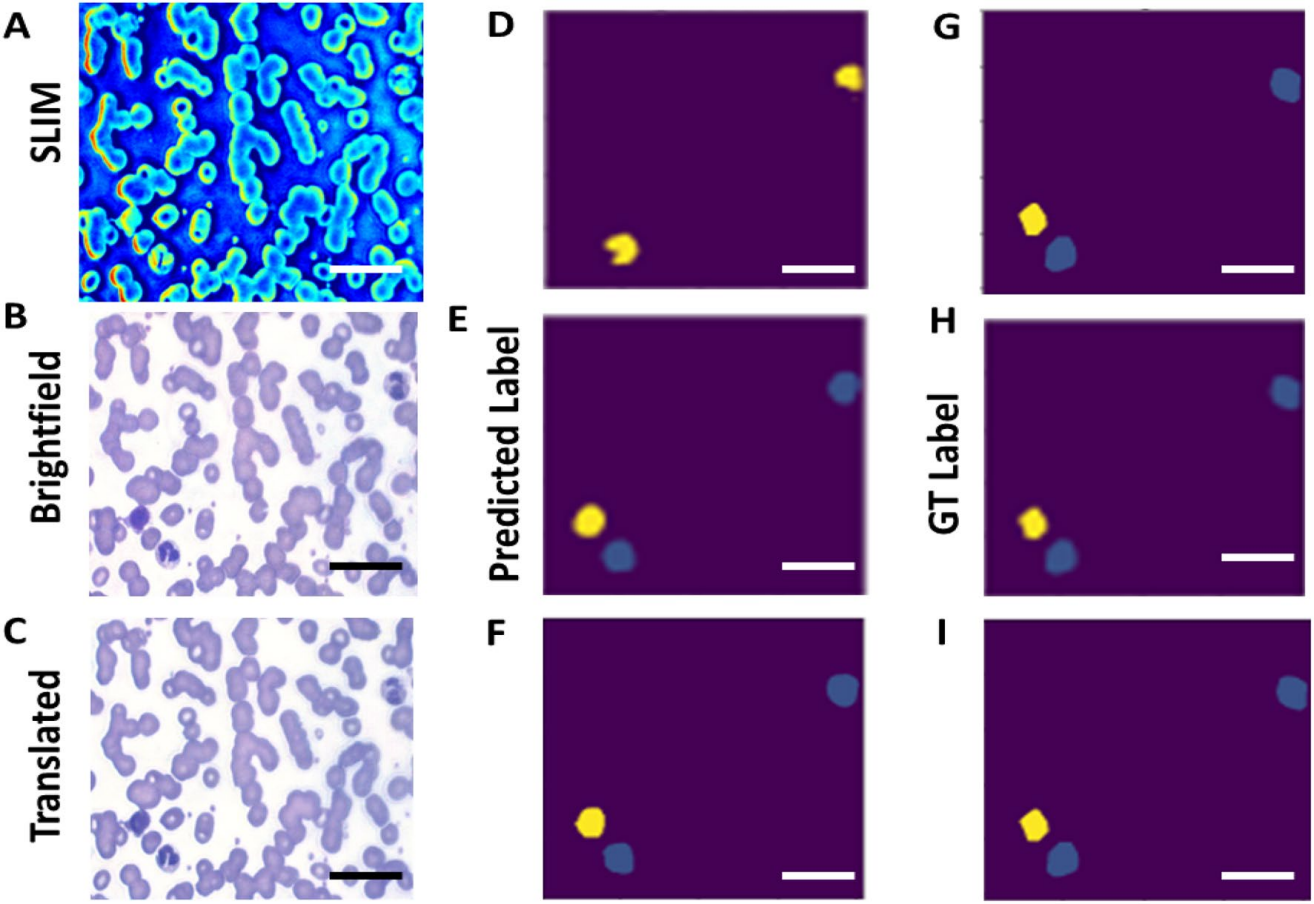
Semantic segmentation results. (**A**) Example SLIM input, (**B**) example brightfield input, and (**C**) example translated input. (**D–F**) Corresponding predicted labels, and (**G–I**) corresponding ground truth labels. Scale bars; 20 µm.

An example of a WBC segmentation is shown in Fig. 6 for SLIM, brightfield and translated cases. In this example there is a neutrophil in the top right corner, one in the bottom left corner, and a lymphocyte in the bottom left corner. Figure 6D–F are the predicted labels from the model, and Fig. 6G–I are the ground truth, with further examples presented in Fig. S3. Both brightfield and translated images have predicted labels similar to the ground truth, with all three cells correctly identified.

## Discussion

Here, we present evidence that our method of combining AI with color spatial light interference microscopy (cSLIM) can quickly identify different white blood cells, such as neutrophils, monocytes, lymphocytes and eosinophils, without manual analysis. This is an important contribution to blood smear analysis, especially given the significance and multitude of leukocyte complications. We demonstrated that applying AI to cSLIM images delivers excellent performance in first artificially generating brightfield micrographs and afterwards localizing and classifying different WBCs. The results for all four categories indicate that the proposed method may be useful in quick screenings for cases of suspected leukocyte disorders.

Not only does this technique offer automatic screening, but multiple blood smear slides can be evaluated rapidly as the overall throughput of the cSLIM scanner is comparable with that of commercial whole slide scanners. Inferring additional information through digital staining would be one way to improve upon these results other than simply adding more images, while keeping the samples label-free. This has recently been accomplished with phase images^54^, RI tomography^55^, and autofluorescence^56^. In our case, artificially recreating various fluorescent tags to identify specific components of white blood cells, such as a molecular tag for human neutrophil elastase (HNE), could help enhance our results. Future scope includes evaluating more images with sufficient instances of basophils and bands to add to the current WBC categories list, as well as imaging blood smears with specific leukocyte abnormalities, such as autoimmune neutropenia and leukemia.

## Supplementary Information

Supplementary Information.

## Data Availability

All data required to reproduce the results can be obtained from the corresponding author upon a reasonable request.

## Code availability

All the code required to reproduce the results can be obtained from the corresponding author upon a reasonable request.

## References

[1] Blumenreich, M.S. *The white blood cell and differential count.* Clinical Methods: The History, Physical, and Laboratory Examinations. 3rd edition, (1990).

[2] Bacusmber JW, Gose EE (1972). Leukocyte pattern recognition. IEEE Trans. Syst. Man Cybern..

[3] Nassar M (2019). Label-free identification of white blood cells using machine learning. Cytometry A.

[4] Spitzer MH, Nolan GP (2016). Mass cytometry: Single cells, many features. Cell.

[5] Lippeveld M (2020). Classification of human white blood cells using machine learning for stain-free imaging flow cytometry. Cytometry A.

[6] Popescu, G., *Quantitative phase imaging of cells and tissues*. (McGraw Hill Professional, 2011).

[7] Hu C, Popescu G (2018). Quantitative phase imaging (QPI) in neuroscience. IEEE J. Sel. Top. Quantum Electron..

[8] Li Y (2019). Quantitative phase imaging reveals matrix stiffness-dependent growth and migration of cancer cells. Sci. Rep..

[9] Majeed H (2017). Quantitative phase imaging for medical diagnosis. J. Biophotonics.

[10] Nguyen TH (2014). Quantitative phase imaging with partially coherent illumination. Opt. Lett..

[11] Sridharan S (2015). Prediction of prostate cancer recurrence using quantitative phase imaging. Sci. Rep..

[12] Takabayashi M (2018). Disorder strength measured by quantitative phase imaging as intrinsic cancer marker in fixed tissue biopsies. PLoS ONE.

[13] Nguyen TH (2017). Gradient light interference microscopy for 3D imaging of unlabeled specimens. Nat. Commun..

[14] Kandel ME (2019). Epi-illumination gradient light interference microscopy for imaging opaque structures. Nat. Commun..

[15] Kandel ME (2018). Real-time halo correction in phase contrast imaging. Biomed. Opt. Express.

[16] Popescu G (2008). Optical imaging of cell mass and growth dynamics. Am. J. Physiol. Cell Physiol..

[17] Ferraro P (2006). Quantitative phase-contrast microscopy by a lateral shear approach to digital holographic image reconstruction. Opt. Lett..

[18] Eldridge WJ, Hoballah J, Wax A (2018). Molecular and biophysical analysis of apoptosis using a combined quantitative phase imaging and fluorescence resonance energy transfer microscope. J. Biophotonics.

[19] Park HS (2019). Quantitative phase imaging of erythrocytes under microfluidic constriction in a high refractive index medium reveals water content changes. Microsyst. Nanoeng..

[20] Eldridge WJ (2019). Shear modulus measurement by quantitative phase imaging and correlation with atomic force microscopy. Biophys. J..

[21] Park HS (2018). Invited article: Digital refocusing in quantitative phase imaging for flowing red blood cells. APL Photonics.

[22] Casteleiro Costa P (2020). Noninvasive white blood cell quantification in umbilical cord blood collection bags with quantitative oblique back-illumination microscopy. Transfusion.

[23] Ledwig P, Robles FE (2021). Quantitative 3D refractive index tomography of opaque samples in epi-mode. Optica.

[24] Robles, F.E. *Epi-mode tomographic quantitative phase imaging in thick scattering samples*. In *Label-free Biomedical Imaging and Sensing (LBIS) 2020*. International Society for Optics and Photonics (2020). 10.1364/BOE.10.003605 PMC6640824 31360607

[25] Kemper B (2011). Simplified approach for quantitative digital holographic phase contrast imaging of living cells. J. Biomed. Opt..

[26] Park Y, Depeursinge C, Popescu G (2018). Quantitative phase imaging in biomedicine. Nat. Photonics.

[27] Di Caprio G (2010). Quantitative label-free animal sperm imaging by means of digital holographic microscopy. IEEE J. Sel. Top. Quantum Electron..

[28] Marquet P (2005). Digital holographic microscopy: A noninvasive contrast imaging technique allowing quantitative visualization of living cells with subwavelength axial accuracy. Opt. Lett..

[29] Lee K (2013). Quantitative phase imaging techniques for the study of cell pathophysiology: From principles to applications. Sensors.

[30] Jin D (2017). Tomographic phase microscopy: Principles and applications in bioimaging. JOSA B.

[31] Wang Z (2011). Spatial light interference microscopy (SLIM). Opt. Express.

[32] Majeed, H. *et al.**Quantitative histopathology of stained tissues using color spatial light interference microscopy (cSLIM).* Sci. Rep. **9** (2019). 10.1038/s41598-019-50143-x PMC6789107 31604963

[33] Fanous M (2020). Quantifying myelin content in brain tissue using color spatial light interference microscopy (cSLIM). PLoS ONE.

[34] Rivenson, Y. *et al.*, *PhaseStain: The digital staining of label-free quantitative phase microscopy images using deep learning.* Light: Sci. Appl., **8**(1): 23 (2019). 10.1038/s41377-019-0129-y PMC6363787 30728961

[35] de Haan K (2020). Automated screening of sickle cells using a smartphone-based microscope and deep learning. NPJ Digital Med..

[36] Subramanian, S. *et al.**MedICaT: A Dataset of Medical Images, Captions, and Textual References.* arXiv preprint arXiv:2010.06000, (2020).

[37] MacAvaney, S. *et al.**Ranking significant discrepancies in clinical reports*. In *European Conference on Information Retrieval*. (Springer, 2020).

[38] Kohlberger, T. *et al.**Whole-slide image focus quality: Automatic assessment and impact on ai cancer detection.* J. Pathol. Inform. **10** (2019). 10.4103/jpi.jpi_11_19 PMC6939343 31921487

[39] Krause J (2018). Grader variability and the importance of reference standards for evaluating machine learning models for diabetic retinopathy. Ophthalmology.

[40] Poplin, R. *et al.**Predicting cardiovascular risk factors from retinal fundus photographs using deep learning. arXiv 2017.* arXiv preprint arXiv:1708.09843. 10.1038/s41551-018-0195-0 31015713

[41] Liu, Y. *et al.**Detecting cancer metastases on gigapixel pathology images.* arXiv preprint arXiv:1703.02442, (2017).

[42] Zhang JK (2020). Label-free colorectal cancer screening using deep learning and spatial light interference microscopy (SLIM). APL Photonics.

[43] Hou, L. *et al.**Patch-based convolutional neural network for whole slide tissue image classification*. In *Proceedings of the IEEE Conference on Computer Vision and Pattern Recognition* (2016). 10.1109/CVPR.2016.266 PMC5085270 27795661

[44] Kandel ME (2020). Phase imaging with computational specificity (PICS) for measuring dry mass changes in sub-cellular compartments. Nat. Commun..

[45] Goswami, N., *et al.**Rapid SARS-CoV-2 Detection and Classification Using Phase Imaging with Computational Specificity.* bioRxiv, (2020). 10.1038/s41377-021-00620-8 PMC8408039 34465726

[46] Goswami, N., *et al.**Single virus detection using phase imaging with computational specificity (PICS)*. In *Quantitative Phase Imaging VII*. International Society for Optics and Photonics (2021).

[47] Isola, P., *et al.**Image-to-image translation with conditional adversarial networks*. In *Proceedings of the IEEE conference on computer vision and pattern recognition* (2017).

[48] de Haan K (2021). Deep learning-based transformation of H&E stained tissues into special stains. Nat. Commun..

[49] Zhang, Z. *Improved adam optimizer for deep neural networks*. In *2018 IEEE/ACM 26th International Symposium on Quality of Service (IWQoS)*. IEEE (2018.).

[50] Tan, M., Pang, R. and Le, Q.V. *Efficientdet: Scalable and efficient object detection*. In *Proceedings of the IEEE/CVF Conference on Computer Vision and Pattern Recognition* (2020).

[51] Deng, J., *et al.**Imagenet: A large-scale hierarchical image database*. In *2009 IEEE Conference on Computer Vision and Pattern Recognition*. IEEE (2009).

[52] Hutchison, R.E. and Schexneider, K. *Leukocytic disorders.* Henry’s Clinical Diagnosis and Management by Laboratory Methods. Philadelphia: (Saunders Elsevier, 2011).

[53] Chernecky, C. and B. Berger, *Differential leukocyte count (diff)-peripheral blood.* Laboratory tests and diagnostic procedures, 440–446 (2013).

[54] Kandel, M. Phase Imaging with Computational Specificity (PICS) for measuring dry mass changes in sub-cellular compartments. *Nat. Commun.* (2020). 10.1038/s41467-020-20062-x PMC7721808 33288761

[55] Jo, Y., *et al.*, *Data-Driven Multiplexed Microtomography of Endogenous Subcellular Dynamics.* bioRxiv, (2020).

[56] Rivenson Y (2019). Virtual histological staining of unlabelled tissue-autofluorescence images via deep learning. Nat Biomed. Eng..

