# Supplementary material for: White blood cell detection, classification and analysis using phase imaging with computational specificity (PICS)
Source: Sci Rep. 2022 Nov 21;12:20043. doi: 10.1038/s41598-022-21250-z (PMC9681839; doi:10.1038/s41598-022-21250-z)
Supplement: Supplementary file 1 — Supplementary Information. [file 41598_2022_21250_MOESM1_ESM.pdf]

White blood cell detection, classification and analysis using phase imaging with computational specificity (PICS)

Supplementary Information

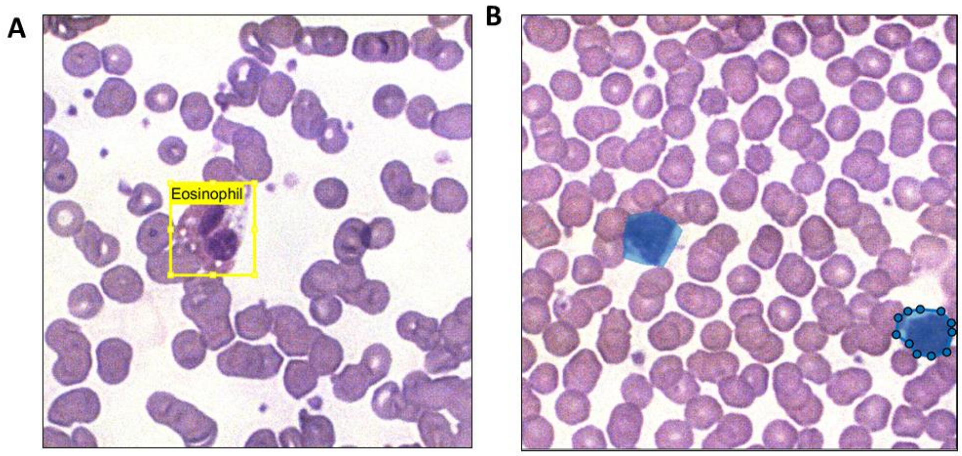

Fig. S1. Labelling examples using the MATLAB imageLabeler for both (A) rectangle (in this case of an eosinophil) and (B) pixel labels (in this case of two lymphocytes).

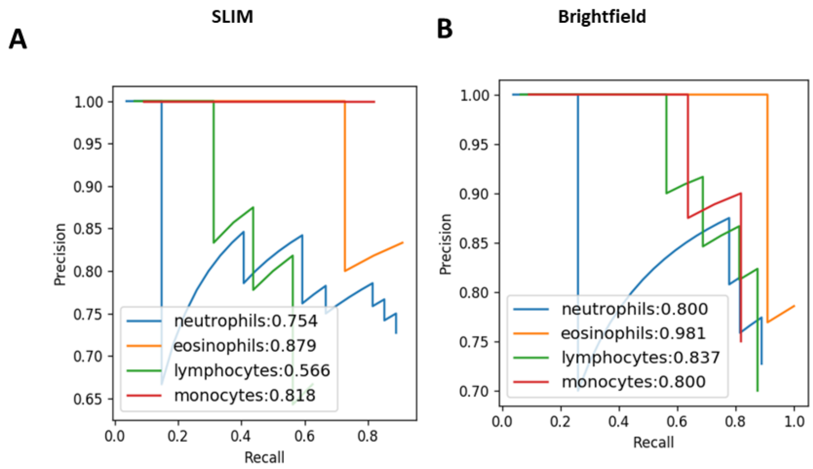

Fig. S2. Precision-recall curves for SLIM (A), and brightfield (B) images.

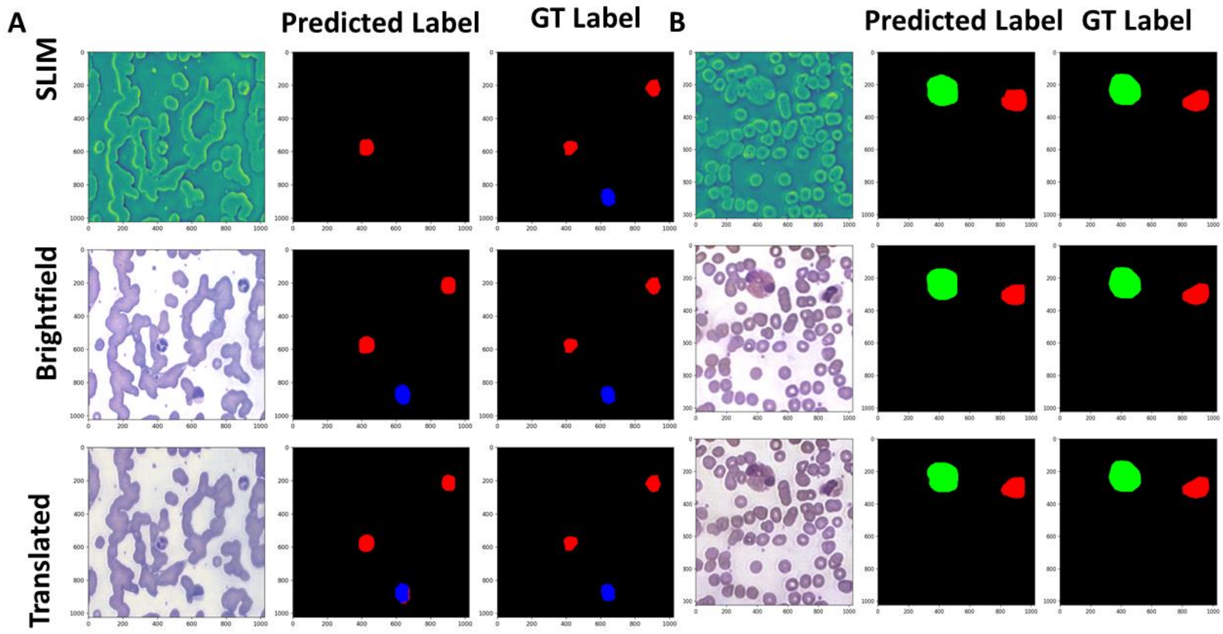

Fig.S3. Prediction examples for a frame with (A) two neutrophils(red) and a lymphocyte (blue) and (B) a frame with an eosinophil (green) and a lymphocyte (red).
